# Effect of Temperature, Relative Humidity, and Incubation Time on the Mycotoxin Production by *Fusarium* spp. Responsible for Dry Rot in Potato Tubers

**DOI:** 10.3390/toxins16100414

**Published:** 2024-09-24

**Authors:** Maria Gutiérrez-Pozo, Carol Verheecke-Vaessen, Sofia Kourmpetli, Leon A. Terry, Angel Medina

**Affiliations:** 1 Magan Centre of Applied Mycology, Cranfield University, Cranfield MK43 0AL, UK; m.gutierrez.pozo.19@gmail.com (M.G.-P.); c.verheecke@cranfield.ac.uk (C.V.-V.); 2 Plant Science Laboratory, Cranfield University, Cranfield MK43 0AL, UK; s.kourmpetli@cranfield.ac.uk (S.K.);

**Keywords:** ecophysiology, T-2, HT-2, fungal growth, potato storage

## Abstract

Potato is the fourth most consumed crop in the world. More than half of the crop is stored for three to nine months at cold temperatures (3–10 °C) for the fresh and seed market. One of the main causes of fresh potato waste in the retail supply chain is the processing of fungal and bacterial rots during storage. Dry rot is a fungal disease that mainly affects the potato crop during storage and is responsible for 1% of tuber losses in the UK. It is produced by *Fusarium* spp., such as *Fusarium sambucinum* and *F. oxysporum*, which can lead to the accumulation of mycotoxins in the potato tuber. Little is known about the impact of environmental factors on the accumulation of mycotoxins in potato tubers. Understanding the ecophysiology of these fungi is key to mitigating their occurrence under commercial storage conditions. Therefore, this work aimed to elucidate the effect of three different temperatures (5, 10, and 15 °C) and two different water activities (a_w_; 0.97, 0.99) on the ecophysiology and mycotoxin accumulation of *F. sambucinum* and *F. oxysporum* in a potato-based semi-synthetic medium. The mycotoxin accumulation was then studied *in vivo*, in potato tubers cultivated under organic farming conditions, stored for 40 days at 8.5 °C. Results showed that higher temperatures and a_w_ enhanced fungal growth, lag time, and mycotoxin accumulation *in vitro*. Growth rate was 2 and 3.6 times higher when the temperature increased from 5 to 10 and 15 °C, respectively. Six different mycotoxins (T-2, HT-2, diacetoxyscirpenol, 15-acetoxyscirpenol, neosolaniol, and beauvericin) were detected *in vitro* and *in vivo*. T-2 was the most abundant mycotoxin detected *in vitro*, observing 10^6^ ng of T-2/g media after 21 days of incubation at 10 °C and 0.99 a_w_. Due to the long period of time that potato tubers spend in storage, the fluctuations of environmental factors, such as temperature and relative humidity, could promote the development of fungal rot, as well as mycotoxin accumulation. This could result in important food and economic losses for the potato market and a threat to food safety.

## 1. Introduction

Potato (*Solanum tuberosum* L.) is the fourth main food crop in the world after maize, rice, and wheat, and is widely consumed every year with a total production of 370 million tons in 2023 [1]. Potato susceptibility to fungal or bacterial diseases varies across potato cultivars. One of the main factors is the periderm thickness of the tubers [2]. During postharvest storage, more than half of the potato crop is stored for three to nine months at 4–10 °C for the fresh and processing markets and at temperatures below 4 °C for the seed market. During the storage period, the appearance of fungal or bacterial rots is a challenge that must be faced by the potato industry. The main fungal disease in Europe after black dot is dry rot, causing postharvest fungal decay and therefore consequently large economic losses [3]. Currently, 17 different species and five variants of *Fusarium* have been identified as responsible for dry rot in potato tubers, including *F. avenaceum*, *F. solani* var. *coeruleum*, *F. sambucinum*, and *F. oxysporum*, among others [3,4]. *Fusarium* spp. generally infect potato tubers through surface wounds or natural openings at pre- and postharvest stages, resulting in wrinkled brown skin and sunken tissue with a dry appearance [5].

Therefore, maintaining the optimal environmental conditions (temperature and relative humidity) during storage is essential for controlling the incidence of fungal or bacterial rots. Understanding the *in vitro* ecophysiology of *Fusarium* spp. responsible for these diseases will provide insight into how different environmental conditions affect the development of dry rot in potato tubers during storage. To determine the effect of different relative humidity on the development of *Fusarium* spp. *in vitro*, a range of water activities (a_w_) can be used. The a_w_ is related to the water available in the matrix for fungal growth. It is considered one of the most important factors affecting fungal growth and their secondary metabolites production [6,7,8,9]. Therefore, this relationship will allow the simulation of a constant relative humidity in commercial storage facilities at a specific cold temperature.

Some of the *Fusarium* spp., such as *F. sambucinum*, *F. sulphureum*, *F. coeruleum*, *F. graminearum*, and *F. oxysporum*, responsible for dry rot in potato tubers can produce secondary metabolites known as mycotoxins. *Fusarium* spp. produce both trichothecenes and non-trichothecenes [3]. Trichothecenes are classified into four types (A, B, C, and D) based on their chemical structure, with types A and B being detected in rotten potato tubers [10]. T-2 toxin, HT-2 toxin, neosolaniol (NEO), diacetoxyscirpenol (DAS), and 15-acetoxyscirpenol (15-AS) are type A trichothecenes, while nivalenol, fusarenon X, deoxynivalenol (DON), 3-Acetyldeoxynivalenol (3-ADON), and 15-acetyldeoxynivalenol (15-ADON) are considered trichothecenes type B. Ingestion of trichothecenes can cause serious health issues in animals and humans, presenting immunosuppressive and mutagenic effects [10].

In previous studies, fusarenon X, 3-ADON, T-2, and DAS were detected in dry-rot-affected potato tubers infected with *F. sulphureum*, *F. solani*, and *F. sambucinum* at different storage temperatures (5 °C, 20 °C) in two potato cultivars [11,12]. *F. graminearum* was also identified as a producer of DON, nivalenol, 3-ADON, and 15-ADON in inoculated potato tubers [13].

The main non-trichothecenes are beauvericin, enniatin, fumonisin B1, fumonisin B2, fusarin C, zearalenone (ZEN), and fusaric acid; they all have been previously detected in potatoes infected with *F. oxysporum* [14].

Although previous studies have evaluated the effect of temperature and a_w_ in some *Fusarium* spp., different temperatures were studied. In the present study, the effect of these environmental factors was evaluated *in vitro* and *in vivo*, not only on the fungal development, but also in their mycotoxin accumulation. There is limited research on the accumulation of mycotoxins in potato tubers stored under different environmental conditions. Therefore, elucidating the effect of storage temperature and relative humidity on the accumulation of mycotoxins will provide insight into the food safety of potato tubers. Besides, the time that potato tubers spend in storage, previous to their release to the market, could have an effect on the disease severity, and consequently on the mycotoxin accumulation.

The aims of this study were to (1) elucidate the effect of three different temperatures (5, 10, and 15 °C) and two different a_w_ (0.97, 0.99) on the ecophysiology of *F. sambucinum* and *F. oxysporum* in a potato-based semi-synthetic media; (2) evaluate the effect of temperature and a_w_ on the mycotoxin accumulation *in vitro* of both species; and (3) determine the mycotoxin accumulation in potato tubers under standard commercial storage conditions.

## 2. Results

### 2.1. Effect of Temperature and a_w_ on Two Fusarium spp. Growth Parameters on Potato-Based Media

**Figure 1:**
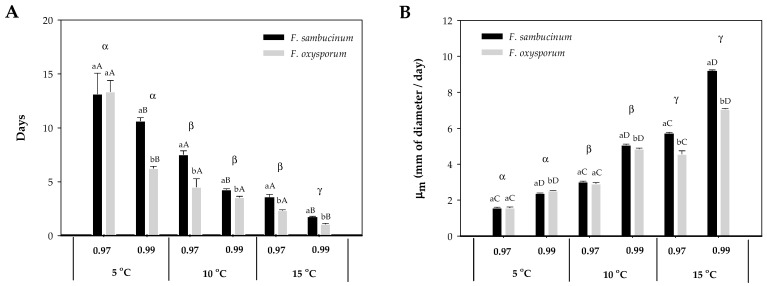
Effect of temperature (5, 10, 15 °C) and a_w_ (0.97, 0.99) on *F. sambucinum* and *F. oxysporum* growth. (**A**) Lag time (λ) and (**B**) growth rate (µ_m_) of both *Fusarium* spp. on NPDA. Data show the means of six replicates ± standard deviation. a, b: Significant differences between *Fusarium* spp. at each specific a_w_ and temperature. Different capital letters: Significant differences between a_w_ at each temperature for each species. α, β, Ɣ: Significant differences between temperatures for each specific *Fusarium* spp. and a_w_ (*t*-test, *p*-value < 0.05).

The increase in temperature from 5 to 15 °C resulted in a significant decrease in the lag time (λ) of both *Fusarium* spp. (*F. sambucinum*, *F. oxysporum*) (Figure 1A). At the lowest temperature, 5 °C, different a_w_ did not affect the λ of either *Fusarium* spp. At 10 °C, a_w_ only significantly affected *F. sambucinum* (*p*-value < 0.007), with a shorter λ at the highest a_w_ (0.99). Similar results were obtained at the highest temperature, where significantly shorter λ were observed for both *Fusarium* spp. and a_w_ (*p*-values < 0.004). The effect of temperature on λ of both *Fusarium* spp. differed between a_w_; at 0.97 a_w_, significant differences were only detected between 5 and 15 °C and 10 and 15 °C (*p*-values < 0.008). While at 0.99 a_w_, significant differences (*p*-values < 0.006) were detected between the three temperatures.

Differences in the λ were observed between both *Fusarium* spp.; significant differences were detected at the highest a_w_ (0.99) at 5 °C, and at both a_w_ at 10 and 15 °C (*p*-values < 0.007). *F. sambucinum* presented generally a higher lag time than *F. oxysporum*.

The effect of temperature and a_w_ on both *Fusarium* species’ growth rate (µ_m_) is presented in Figure 1B. The higher growth rate was achieved by *F. sambucinum* at 15 °C and 0.99 a_w_, with 9 mm of diameter per day. Overall, there was an increase in growth rate with temperature and a_w_. Significant differences (all *p*-values < 0.005) were detected for each of the *Fusarium* spp. between a_w_ (0.97 and 0.99) at the three different temperatures (5, 10, and 15 °C) with a higher growth rate at the highest a_w_ (0.99). Significant differences (*p*-values < 0.0001) in the growth rate of both *Fusarium* spp. were detected between the three different temperatures, with a higher growth rate at the highest temperature (15 °C).

When growth rate was compared between *Fusarium* spp., significant differences (*p*-values < 0.003) were only detected at the highest a_w_ (0.99) at 5 and 10 °C, while at 15 °C, significant differences were detected at both a_w_ (0.97, 0.99). At 5 °C × 0.99 a_w_, *F. oxysporum* presented a higher growth rate when compared with *F. sambucinum*, while at 10 °C × 0.99 a_w_ and 15 °C at both a_w_, *F. sambucinum* grew faster.

### 2.2. Effect of Cultivar and Stage of Storage of Potato Tubers on the External Lesion Caused by F. sambucinum

**Figure 2:**
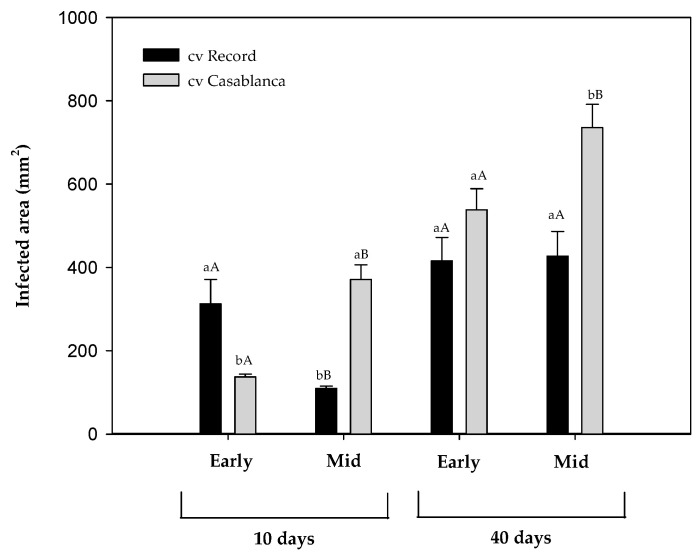
Infected area (mm^2^) of *Fusarium sambucinum* on two different cultivars of potato tubers (cv. Record and cv. Casablanca) that were previously stored for 10 (Early) and 22 weeks (Mid) after 10 and 40 days at 8.5 °C. Data show the means of three replicates ± standard deviation. a, b: Significant differences between cultivars at each specific time and stage of storage. A, B: Significant differences between stages of the storage (early and mid) of potato tubers at each specific time and cultivar (Kruskal–Wallis, *p*-value < 0.05).

Two potato cultivars (cv. Record; cv. Casablanca), previously stored for 10 (early-stage) and 22 weeks (mid-stage) were inoculated with *F. sambucinum* and stored for 40 days at 8.5 °C. The external lesions were evaluated after 10 and 40 days of incubation at cold temperature and presented as an infected area (mm^2^) in Figure 2.

At the early storage stage, after 10 days of post-inoculation cold incubation, significant differences (*p*-value < 0.05) in the infected area were observed between cultivars, with cv. Record being more affected by *F. sambucinum* than cv. Casablanca. At the same stage, 40 days after inoculation, no significant differences were detected between cultivars.

At the mid-stage of storage, cv. Casablanca presented a significantly (*p*-value < 0.05) higher infected area than cv. Record at 10 and 40 days after inoculation. The stage of the storage of potato tubers reduced infection in cv. Record at an early the stage only but significantly increased the infection area of cv. Casablanca (*p*-value < 0.05) at both storage stages.

### 2.3. Effect of Temperature and a_w_ on Mycotoxin Accumulation in vitro

#### 2.3.1. Trichothecenes Accumulation

**Figure 3:**
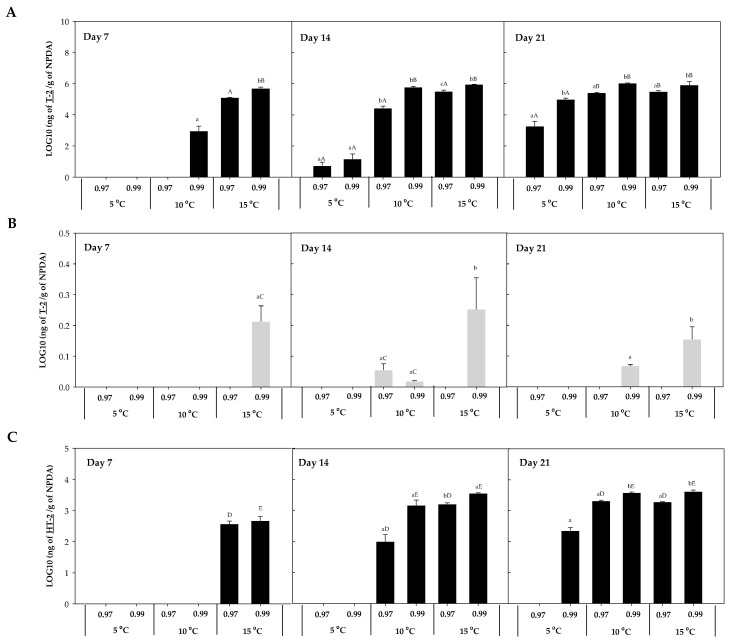
T-2 accumulation in presence of *F. sambucinum* (**A**) and *F. oxysporum* (**B**) and HT-2 accumulation in presence of *F. sambucinum* (**C**) after 7, 14, and 21 days of incubation at different environmental conditions (temperature and water activity). Data show the means of six replicates ± standard deviation. a–c: Significant differences between temperatures at each specific a_w_. Different capital letters:: Significant differences between a_w_ at each specific temperature (*t*-test, *p*-values < 0.05).

The mycotoxin accumulation on NPDA was studied after 7, 14, and 21 days. Five trichothecenes A, known to be produced by *Fusarium* spp., were detected in the present work (T-2, HT-2, DAS, 15-AS, and NEO). No trichothecenes B were detected in any of the samples analysed. T-2, HT-2, DAS, 15-AS, and NEO were detected in the presence of *F. sambucinum*, while in the presence of *F. oxysporum* only T-2 was detected. The accumulation of each of these five trichothecenes was studied at the three different temperatures (5, 10, 15 °C) and two different a_w_ (0.97, 0.99). T-2 and HT-2 accumulation for *F. sambucinum* and T-2 accumulation for *F. oxysporum* are presented in Figure 3.

T-2 concentration in the presence of *F. sambucinum* ranged from not detected to 10^6^ ng/g in NPDA (Figure 3A), with significantly higher (*p*-values < 0.05) concentrations at 0.99 a_w_ compared to 0.97 a_w_. Temperature significantly (*p*-values < 0.05) affected the accumulation of T-2 in *F. sambucinum*. After 7 days of incubation, its presence was only observed at the highest temperature, while after 14 days it was also detected at 10 °C. Besides, at the end of the incubation period it was higher at 10 °C and detected at the lowest temperature (5 °C) as well.

The accumulation of T-2 in the presence of *F. oxysporum* (Figure 3B) followed a similar tendency, although the levels detected were 10^6^ times lower than the ones found in *F. sambucinum* and some values were below the Limit of Detection (LOD). In presence of *F. oxysporum*, T-2 concentrations ranged from not detected to 60 ng/g in NPDA. T-2 was the only mycotoxin detected in presence of *F. oxysporum* over all the different combinations of temperature and a_w_ tested.

The accumulation of HT-2 in *F. sambucinum* cultures (Figure 3C) followed a similar tendency to T-2, although its concentration was 10^3^ time lower than T-2. After 7 days of incubation, 400.73 ± 30.42 and 547.87 ± 23.21 ng of HT-2/g NPDA were detected at 15 °C and 0.97 and 0.99 a_w_, respectively. While after 14 days of incubation, HT-2 detection was also observed at 10 °C, and was significantly higher (*p*-values < 0.05) at 15 °C (compared to 10 °C). At the end of the incubation period, HT-2 was also detected at 5 °C (225.25 ± 55.51 ng HT-2/g NPDA). Significant differences (*p*-values < 0.05) were only detected at 0.99 a_w_ between 5 and 10 °C, while no differences were observed after 21 days of incubation between 10 and 15 °C. Overall, the a_w_ of the media significantly affected the HT-2 accumulation; at the highest a_w_, the concentration was higher.

**Figure 4:**
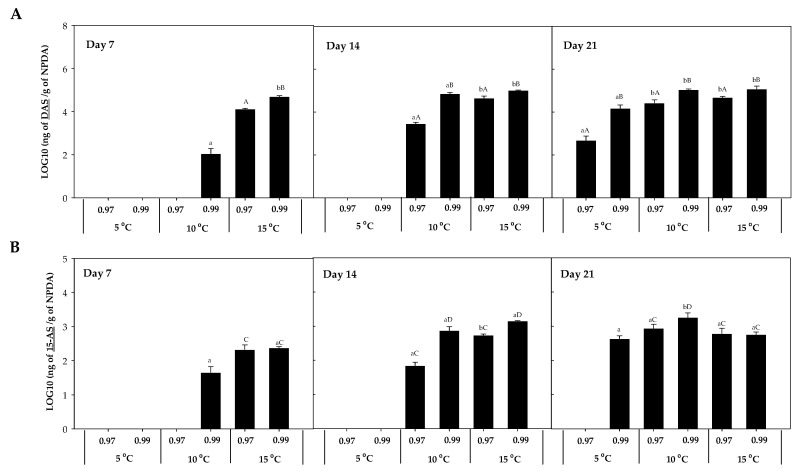
Diacetoxyscirpenol (DAS) (**A**) and 15-acetoxyscirpenol (15-AS) (**B**) accumulation in the presence of *F. sambucinum* after 7, 14, and 21 days of incubation in different environmental conditions (temperature and water activities). Data show means of six replicates ± standard deviation. a, b: Significant differences between temperatures at each specific a_w_. Different capital letters:: Significant differences between a_w_ at each specific temperature (*t*-test, *p*-values < 0.05).

The accumulation of DAS was 10^3^ times higher than 15-AS, observing the highest concentration at 10 °C × 0.99 a_w_ (1807.2 ± 55.51 ng HT-2/g NPDA; Figure 4). After 7 days of incubation, their presence was detected at 15 °C, except for 15-AS which was also detected at 10 °C × 0.99 a_w_ (Figure 4B). After 14 days, their detection was also predominant at 10 and 15 °C, and after 21 days, their presence was detected in almost all the conditions, with the lowest concentration detected at 5 °C × 0.97 a_w_. Significant differences (*p*-values < 0.05) were detected between temperatures (5, 10, and 15 °C) for these two mycotoxins (DAS and 15-AS). The concentration of DAS after 14 and 21 days of incubation was significantly higher at the highest water activity (0.99) compared to 0.97 a_w_ (*p*-values < 0.05) (Figure 4A).

**Figure 5:**
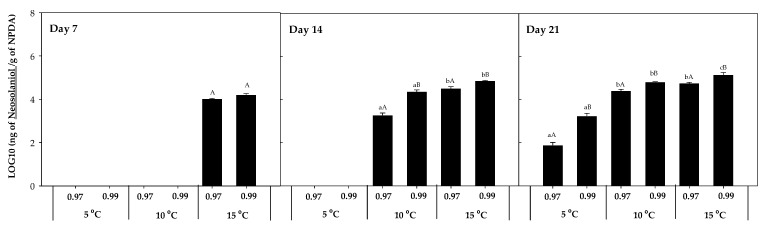
Neosolaniol (NEO) accumulation in the presence of *F. sambucinum* after 7, 14, and 21 days of incubation in different environmental conditions (temperature and water activities). Data show means of six replicates ± standard deviation. a–c: Significant differences between temperatures at each specific a_w_. A, B: Significant differences between a_w_ at each specific temperature (*t*-test, *p*-values < 0.05).

NEO was also detected in presence of *F. sambucinum* (Figure 5), and its accumulation was mainly detected at 10 and 15 °C, independently of the time of incubation. Its presence at 5 °C was only detected at the highest a_w_ after 21 days of incubation. The highest concentration of NEO was detected after 21 days of incubation at the highest temperature and 0.99 a_w_ (1.45·105 ± 2.31·103 ng NEO/g NPDA). Significantly higher (*p*-values < 0.05) accumulation was detected with higher temperatures and water activities.

#### 2.3.2. Other Mycotoxins Accumulation

**Figure 6:**
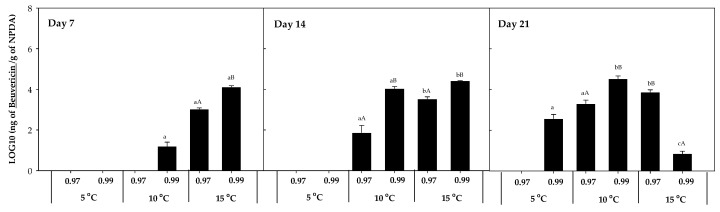
Beauvericin accumulation in presence of *F. sambucinum* after 7, 14, and 21 days of incubation in different environmental conditions (temperature and water activities). Data show means of six replicates ± standard deviation. a–c: Significant differences between temperatures at each specific a_w_. A, B: Significant differences between a_w_ at each specific temperature (*t*-test, *p*-values < 0.05).

The accumulation of other mycotoxins, such as beauvericin, fumonisin B1 and B2, fusaric acid, and ZEN, was also evaluated. However, beauvericin was the only mycotoxin detected in the presence of *F. sambucinum*, with no additional mycotoxins detected in the presence of *F. oxysporum*. The accumulation of beauvericin (Figure 6) was significantly affected by the a_w_ of the media. After 7 days of incubation, it was mainly detected at 15 °C × 0.99 a_w_ (1.35 × 10^4^ ± 2.00 × 10^2^ ng beauvericin/g NPDA), while after 14 days of incubation it was detected at 10 and 15 °C at the highest a_w_ (0.99). After 21 days, its detection was significantly higher (*p*-values < 0.04) at 10 °C × 0.99 a_w_ and was detected at 5 °C but not at 15 °C × 0.99 a_w_.

### 2.4. Effect of Cultivar and Stage of the Storage on Mycotoxin Accumulation on Potato Tubers Inoculated with Fusarium sambucinum

The mycotoxin accumulation on potato tubers inoculated with *F. sambucinum* was studied on two different cultivars (cv. Record, cv. Casablanca) after 10 and 40 days of incubation at 8.5 °C in potato tubers that had been stored for 10 (early-stage) and 22 weeks (mid-stage) prior to inoculation. Six mycotoxins (T-2, HT-2, DAS, 15-AS, NEO, and beauvericin), known to be produced by *Fusarium* spp., were detected in both cultivars. As it can be observed in Table 1, the highest accumulation of mycotoxins was detected in potato tubers from a mid-stage. No significant differences (*p*-values < 0.05) were detected between cultivars after 10 days of incubation at the early stage of storage, except for NEO, where higher accumulation was observed in cv. Record. Although, in general a slightly higher accumulation of mycotoxins was observed in cv. Record compared to cv. Casablanca, the infected area produced by *F. sambucinum* after 10 days of incubation was higher in cv. Record than in Casablanca. The mycotoxin that was detected at the highest concentration was T-2, followed by HT-2, DAS, and 15-AS. Besides, when the effect of the stage of storage of the potato tubers was analysed based on the mycotoxin accumulation after 10 days of incubation, a significant increase in the mycotoxin content was observed for the cv. Casablanca compared to Record. Casablanca potato tubers that were stored for a longer period resulted in a significantly higher content of mycotoxins (*p*-values < 0.05), while this effect was not observed for cv. Record. The mycotoxin content of non-inoculated potato tubers (control) was also evaluated; as expected, no mycotoxins were detected and none of them presented any visible fungal development.

## 3. Discussion

This study has compared the effect of two-way interacting environmental conditions (temperature, a_w_) on *F. sambucinum* and *F. oxysporum* growth and temporal mycotoxin accumulation *in vitro*. It has been previously observed that in commercial potato stores, spatial differences are often observed in temperature and relative humidity conditions [15,16]. Therefore, unveiling how the different environmental conditions can affect fungal development and mycotoxin accumulation of *Fusarium* spp. is essential for the potato industry. Providing farmers with good practices to avoid or reduce the development of dry rot is beneficial for storage management and can contribute to the reduction of potato losses, while maintaining high food safety standards.

In this study, both *Fusarium* spp. have shown a reduction in lag time and an increase in growth rate because of increased temperature and a_w_. An increase in temperature modified the overall lag time values from 13.1 to 3.6 days. The growth rate of *F. sambucinum* at 15 °C was 3.6 times higher than at 5 °C, and double that at 10 °C. *F. sambucinum* showed a lower lag time and higher growth rate compared to *F. oxysporum* under the same conditions. *In vivo* studies on the development of *Fusarium* spp., responsible for dry rot in potato tubers, suggested that changes in temperature from 5 °C to 20–25 °C were directly affecting the severity of damage in potato tubers infected with *Fusarium* spp. (*F. coeruleum*, *F. sambucinum*, *F. avenaceum*), responsible for dry rot [17,18,19,20,21,22]. An increase in the severity of dry rot, produced by *F. sambucinum*, has also been observed under high relative humidity conditions [19]. Although these results were observed *in vivo*, they were in accordance with our results. Therefore, an increase in the humidity conditions allowed a quicker development of *Fusarium* spp.

The effect of temperature on the growth of *Fusarium* spp. *in vitro* has been studied several times on different media, such as PDA. Daami-Remadi et al. (2006) studied the effect of temperature on both pathogens, different isolates, *in vivo* and *in vitro*. In their *in vitro* experiment, both *Fusarium* spp. were inoculated in non-modified PDA (0.995 a_w_), and the diameter of colonies was observed six days after incubation at the same temperatures as the present study (5, 10, 15 °C) [23]. The main differences between these results and ours were for *F. oxysporum*. They did not observe any growth at 5 °C and the colony diameter was lower at 10 and 15 °C, by 10 and 15 mm, respectively. Also, they observed larger colonies for *F. sambucinum* compared to *F. oxysporum*; the opposite of what was observed in the present study. Those differences might be due to the different formulation of the media. In our study, a 30%-containing mashed potato media to be more representative of *in vivo* potato tubers. Therefore, the findings achieved in this work suggest that higher temperatures in storage will directly increase the growth of *F. sambucinum* and *F. oxysporum*.

The effect of temperature and a_w_ on *F. sambucinum*, isolated from rice, in rice extract agar (REA) was previously studied by Ferre et al. (2007). The growth rate of *F. sambucinum* at 15 °C at 0.95, 0.98, and 0.995 a_w_ increased along with the a_w_ tested [24]. These data were in accordance with the present study. They observed values between 1 and 3 mm/day at 0.95 and 0.995 a_w_, respectively; values that were almost 1/6 of our results, achieving a growth rate at 15 °C × 0.99 a_w_ of 9 mm/day. This difference might be due to the media and the specific strain of *F. sambucinum*. These results are in accordance with the present findings regarding the effect of the relative humidity on the growth of *F. sambucinum*. The increase in the relative humidity of potato storage rooms will benefit the growth of *F. sambucinum*.

This is the first time where the effect of storage stage of potato tubers has been evaluated on *F. sambucinum* development in two different potato cultivars. A higher lesion severity was observed in cv. Casablanca compared to cv. Record. This severity difference could be because Record is a maincrop cultivar, while Casablanca is an earlier cultivar. This is supported by previous studies where a higher susceptibility to dry rot was observed in earlier cultivars compared to maincrop due to their physiological characteristics [17,21]. This might be caused by the effect of storage time on lesion severity of dry rot. Casablanca, an earlier cultivar, showed higher disease severity with the increase in storage time. This could be due to the physiological changes that the cultivar was experiencing during storage. Although the effect of long-term storage in potato tubers has been previously evaluated in soft rot, a bacterial potato disease [25], there are no studies focused on the effect of dry rot. More recently, the Agriculture and Horticulture Development Board has confirmed that Casablanca is susceptible to both *F. coeruleum* and *F. sulphureum* [26], and our results confirm a higher degree of severity of damage to *F. sambucinum* in cv. Casablanca compared to cv. Record. Our research is the first highlighting studying disease severity of *F. sambucinum* in cv. Record.

Different mycotoxins were detected *in vitro* in the presence of both *Fusarium* spp. The trichothecenes detected in this study were T-2, HT-2, DAS, NEO, and 15-AS, while the only non-trichothecene detected was beauvericin. Elucidating the accumulation of those mycotoxins by *F. sambucinum* and *F. oxysporum*, both responsible for dry rot in potato tubers, at the different environmental conditions studied, can provide interesting information regarding the food safety of potato tubers infected with dry rot. In the presence of *F. sambucinum*, a larger number of mycotoxins were detected: T-2, HT-2, DAS, NEO, 15-AS, and beauvericin. While in presence of *F. oxysporum*, only T-2 was detected. This is in accordance with what has already been reported regarding the mycotoxins produced by both *Fusarium* spp. In presence of *F. oxysporum*, T-2 and beauvericin were detected on different matrixes, while in presence of *F. sambucinum*, T-2, HT-2, NEO, DAS, 15-AS, and beauvericin were detected [27].

In this study, the accumulation of most mycotoxins was directly related to the growth of both *Fusarium* spp. Indeed, an increased growth of the fungi led to a higher accumulation of toxins. Therefore, their production was directly affected by the temperature and a_w_. The accumulation of mycotoxin was also evaluated *in vivo*. T-2, followed by HT-2, DAS, and 15-AS were detected at the highest concentration with a predominance for an increase in mycotoxins presence with time. The same effect as the one observed for the disease severity was observed in cv. Casablanca; those tubers that were stored for a longer time presented a higher accumulation of mycotoxins. Previous research has been carried out on the detection of mycotoxins in potato tubers [10,11,12,13,14,28]. The influence of cultivar and storage temperature on the accumulation of mycotoxins in potato tubers was studied on three *Fusarium* spp. (*F. sambucinum*, *F. solani*, *F. sulphureum*). They evaluated the accumulation of mycotoxins after 21 and 60 days when potato tubers were stored at 20 and 5 °C, respectively. Although their accumulation was higher at 20 °C, after 60 days of storage at 5 °C, Fusarenon X, 3-AcDON, DAS, and T-2 were detected at low concentrations [10]. Their results were in accordance with the results observed here, where a higher concentration of trichothecenes was detected at higher temperatures. However, our study is the first studying the effect of storage time of potato tubers on the mycotoxin accumulation [10,11,12,13,14,28].

The effect of temperature is notable as mycotoxins were already detected at low temperatures (5, 10 °C). This is the first study where mycotoxins, such as T-2, HT-2, DAS, 15-AS, and NEO have been detected in the presence of *Fusarium* spp. responsible for dry rot under cold temperature conditions (below 10 °C). Twenty-one days were needed for both *Fusarium* spp., mainly *F. sambucinum*, to start producing mycotoxins at the lowest temperature studied (5 °C). Therefore, considering that potato tubers can be stored for up to 10 months, if the environmental conditions are optimal for the development of *Fusarium* spp., the accumulation of mycotoxins can take place in the potato tuber. Previous studies have already detected the accumulation of beauvericin in the presence of *F. sambucinum* in MEA and potato tubers, when they were stored at 25 °C. However, DAS was only detected *in vitro*, while its detection was lower than the LOD *in vivo* [29]. This could be due to the differential severity that different potato cultivars present to pathogen infection. Since July 2024, there are EU maximum limits for the sum of T-2 and HT-2 in different commodities, such as cereals. As an example, in maize, the maximum level for direct human consumption is 50 µg/kg [30]. Although there is no current limit for potatoes, a concentration of 3 × 10^4^ and 10^4^ of HT-2 and T-2 ng per g of potato was observed in this study. Considering those values and comparing them with the EU recommendation in maize, they would be exceeding the limit if a regulation is to be imposed. Considering that potato is the fourth most consumed crop in the world, a high concentration of mycotoxins could be present once potato tubers are sold.

These findings were achieved under controlled laboratory conditions, which included surface-sterilisation and space between potato tubers. In a commercial potato storage facility, tubers are stored in bulk in 1-ton boxes, in close contact, where infection between tubers will spread quicker and remain not visible until a significant portion of the box is already infected. This suggests that, not only should there be a regular control of the storage facility, focusing on the sensible areas to changes in temperature, but also new storage systems that allow the early detection of rotten tubers.

Maintaining food safety is our first objective. Therefore, the fluctuation in temperature and relative humidity of potato storage facilities could result in an increase in dry rot in stores, higher severity of the damage appearing in fewer days, and higher accumulation of mycotoxins. Good practices in storage should be reviewed, such as including regular checks for the presence of fungal rots in those areas of the storage where the environmental conditions are more susceptible to change (lower and upper zones). Especially considering that some cultivars, in particular cv. Casablanca, could change their susceptibility to suffer severe damages by dry rot depending on the storage time of potatoes. Those potatoes stored for a longer time, prior to be sold, will present higher severity damages of dry rot as less time will be required for the fungi to infect and higher concentrations of mycotoxins. Furthermore, considering that once the potato tuber arrives to the consumer, the storage temperature will increase and so will the *Fusarium* spp. development and, consequently, the presence of mycotoxins. This will result in consumer exposure to mycotoxins and a large amount of food loss and waste in households, as well as a potential risk to the sustainability of the potato supply chain.

## 4. Conclusions

In this paper, we present evidence that supports the need for data-driven decisions to manage potato storage conditions. Indeed, any increases in temperature and relative humidity in potato storage facilities would result in a higher development of dry rot, and higher accumulation of mycotoxins (T-2, HT-2, DAS, 15-AS, NEO, and beauvericin). Moreover, this work combines *in vitro* and *in vivo* studies showcasing the potential of these pathogens not only to develop under different environmental conditions, but also their potential importance in terms of food safety due to their ability to produce mycotoxins, even at cold temperatures. This is the first study elucidating the accumulation of mycotoxins in the presence of *Fusarium* spp. responsible for dry rot under cold temperatures (5 and 10 °C). These results showed levels of trichothecenes, such as T-2, that are overpassing the new maximum limit in the European Union enforced since July 2024 [30]. Therefore, good practices for potato storage facilities would require an optimal and regular control of potato rots, focusing on storage settings where the environmental conditions are more susceptible to change, such as potato tubers from the lower layer of the bulk where water is accumulated, and higher RH conditions are faced.

Furthermore, it has been demonstrated that Casablanca potato tubers with a longer storage time present severe dry rot symptoms, as well as a higher accumulation of mycotoxins. The potato industry should consider the fact that potato tubers might change their disease severity once infected based on their storage time. Therefore, elucidating the time that each potato cultivar can be stored prior to changing their disease severity could help improve the final quality of potato tubers once they arrive to the consumer. It is especially important considering the long periods of storage and that once the potatoes are taken to retail and to households, where conditions of temperature and water availability are not controlled, these fungal species can develop, produce mycotoxins (e.g., T-2 and HT-2), and be exposed to the consumers. It is important to notice that these molecules can most likely diffuse within the potato tissue. Hence, to avoid any potential food safety problems, the recommendation until more research is developed should be to dispose of any contaminated tubers to avoid the risk of consuming *Fusarium* toxins.

## 5. Materials and Methods

### 5.1. Fungal Pathogens

*Fusarium sambucinum*, responsible for dry rot on potato tubers, was supplied from the Plant Breeding and Acclimatization Institute (IHAR, Błonie, Poland). While *F. oxysporum* was isolated in Bedfordshire, UK from rotten potato tubers cv. Markies in October 2018 and the identification was molecularly confirmed based on Gil-Serna et al. (2016) technique at Cranfield University (UK) [31]. Both fungal strains were subcultured in Potato Dextrose Agar (PDA, Sigma-Aldrich, Dorset, UK) for 7 days at 25 °C in the dark. A glycerol: water (70:30, *v*/*v*) stock solution of the previous culture was prepared separately and stored at -20 °C until its use.

### 5.2. Inoculation and Incubation of F. sambucinum and F. oxysporum In Vitro

Both *Fusarium* spp. were studied *in vitro* in a natural potato-based media at three different temperatures (5, 10, and 15 °C) and at two different water activities (0.97 and 0.99 a_w_). The two different water activities simulated high and low relative humidity conditions, 97% and 99%, respectively.

A semi-synthetic growth medium, Natural Potato Dextrose Agar (NPDA), was formulated to simulate the natural crop commodity. NPDA was prepared with 2% of glucose (Fisher Scientific, Waltham, MA, USA), 1.5% of agar (Sigma-Aldrich, Dorset, UK), and 30% of mashed potato in 1 L of potato infusion. Potato infusion was prepared with 200 g of peeled potatoes (cv. Maris Piper) boiled in 1 L of dH_2_O for 20 min and filtered through a single layer of cheesecloth (Fisher Scientific, USA). After filtration, the glucose and agar were added, as well as the mashed potato from the resulted boiled potatoes. The a_w_ of the resulting solution was then modified with glycerol, based on a previously conducted a_w_ regression line. The a_w_ of the NPDA was measured with a AQUALAB 4TE a_w_ meter (Decagon Instruments; Pullman, Washington, DC, USA). The NPDA media was dispensed in Petri dishes (Ø 9 cm) (ThermoFisher Scientific, Waltham, MA, USA).

An aliquot of the glycerol stock of each of the fungal strains was inoculated on PDA and incubated for 7 days at 25 °C, prior to the experiment. A spore suspension of 10^8^ spores/mL was prepared using a Helber Haemocytometer and 1 µL was plated in the centre of the solid media. NPDA plates were incubated at three temperatures (5, 10, and 15 °C) and two different a_w_ conditions (0.97, 0.99) for 30 days. To maintain the a_w_ of the media, cultures from each pair of conditions (T° × a_w_) were stored inside 12 L airtight boxes containing a solution of glycerol: water adapted to the specific a_w_. Three replicates (12 L airtight boxes) were used per temperature and a_w_ with NPDA non-inoculated plates and inoculated with *F. sambucinum* and *F. oxysporum*.

The mycotoxin content was studied *in vitro* at three different sampling points (7, 14, and 21 days). Three replicates were included and samples were kept at −20 °C until mycotoxin analysis was carried out.

### 5.3. Inoculation and Incubation of F. sambucinum on Potato Tubers

A parallel experiment was carried out *in vivo*, where *F. sambucinum* was inoculated in surface-sterilised potato tubers and incubated at 8.5 °C for 40 days. Two cultivars of potato tubers, cultivated under organic farming conditions, were selected based on their susceptibility to dry rot (cv. Casablanca, cv. Record). Both cultivars were stored for two different periods of time under cold conditions, prior to the experiment. Half were stored for 10 weeks (early stage of storage), and the other half were stored for 22 weeks (mid-stage of storage) at 4 °C.

Potato tubers were washed by soaking them in tap water for five minutes followed by ten minutes in distilled water, removing the excess of soil with a scrubber. Potato tubers were submerged for 15 min in 0.5% NaClO. Afterwards, tubers were washed twice with sterile distilled water and immersed in sterile distilled water for two to three hours before their inoculation. Tubers were wounded (5 mm in diameter) in the centre and inoculated with 200 µL of the spore suspension. Three replicates of twelve potato tubers were stored in 12 L airtight boxes and incubated at 8.5 °C under 98% of relative humidity. Non-inoculated potato tubers were also included as a control with the water/glycerol solution to maintain the relative humidity.

The mycotoxin content of potato tubers was analysed after 10 and 40 days of incubation at 8.5 °C. Three replicates were included per combination of conditions and samples were kept at −20 °C until the analysis was carried out.

### 5.4. Diametric Growth Rates of F. sambucinum and F. oxysporum on Potato-Based Media

Fungal growth was assessed daily after the inoculation from six different plates of each of the different conditions (temperature × a_w_). Colony diameters were measured in two perpendicular directions to each other. Two different growth parameters were calculated, growth rate (µ_m_, mm of diameter per day) and lag time (λ, days), using Microsoft Excel (version 2203). The regression line slope was considered as the growth rate, while the lag time was estimated as the interception between the regression line and the *x*-axis [32].

### 5.5. External Lesion Assessment of Potato Tubers Infected with F. sambucinum

The external lesion was assessed after 10 and 40 days of storage at 8.5 °C. Five potato tubers from each treatment were considered. Assuming the potato lesion to be round, the area of the rots (A) was calculated based on the area of a circle, where *r* was half of the average of the measured diameters of the rot. The area of non-inoculated tubers (control) was subtracted from the area of each rot on each specific day measured.

### 5.6. Mycotoxin Analysis

The mycotoxin content was studied *in vitro* and *in vivo*. The mycotoxin extraction from the semi-synthetic media consisted of three plugs of NPDA of 5 mm in diameter, submerged in Liquid Nitrogen (N_2_) and homogenised with 450–600 µm of glass beads using the Precellys 24 Tissue homogenizer (Bertin Instruments, Montigny-le-Bretonneux, France). For the potato samples, 100 mg of rotten tissue were agitated in a Tissue Homogenizer at 5500 rpm for 20 s followed by a 5 s interval and another 20 s of agitation. Then, a volume of extraction buffer (acetonitrile: water: formic acid, 79:20.9:0.1, *v*/*v*/*v*) was added and they were again homogenised. The volume of the extraction buffer was 4 times the weight of each of the samples. Samples were then left for incubation for 90 min at 25 °C at 300 rpm on a rotary shaker in the dark. Afterwards, the extracts were centrifuged for 10 min at 22,600× *g*, the supernatant (200 µL), transferred to HPLC vials containing 250 µL microinserts (Fisher Scientific, USA), and kept at −20 °C until their analysis.

Ultra-high-performance liquid chromatography tandem mass spectrometry (UHPLC-MS/MS) (Sciex Technologies, Warrington, UK) was used for the mycotoxin detection. Chromatographic separation was achieved on a reverse-phase ACE 3-C_18_ column (2.1 × 100 mm, 3 µm particle size; Hichrom, Reading, UK) equipped with a C_18_ security guard cartridge (4 × 3 mm, Gemini, Agilent Technologies, Santa Clara, CA, USA) kept at 40 °C, as described in Gil-Serna et al. (2022) [33]. The method recovery was validated in potato tissue and NPDA media including the targeted mycotoxins (Supplementary Material). Fifteen mycotoxins were included with standards for the sample analysis. Five trichothecenes A were included: T-2, HT-2, DAS, and 15-AS; five trichothecenes B: DON, 3-ADON, 15-ADON, nivalenol, and fusarenon X; and, five non-trichothecenes mycotoxins: beauvericin, fumonisin B1 and B2, fusaric acid, and ZEN. Multiquant 3.03 software (AB Sciex, Foster City, CA, USA) was used for the analysis of the results.

### 5.7. Statistical Analysis

Statistical analysis was performed using JMP Pro 14 (SAS Institute, INC., Cary, NC, USA). Data sets were tested for normality and homoscedasticity using the Shapiro–Wilk and Levene’s tests, respectively. Those datasets that succeeded in the previous test were analysed using parametric test, ANOVA. A *post hoc* analysis (Tukey–Kramer HSD method) was carried out when significant differences were detected (*p*-value < 0.05). For those datasets that the normality and homoscedasticity test failed, a non-parametric test (Kruskal–Wallis) was performed with a *post hoc* analysis (Wilcoxon test) when significant differences were detected (*p*-value < 0.05).

## Figures and Tables

**Table 1:** Mycotoxin accumulation (ng of toxin/g of potato tuber) in cv. Record and cv. Casablanca potato tubers stored for a short (early-stage) and a long period of time (mid-stage), under cold storage conditions (8.5 °C), 10 and 40 days after inoculation with *F. sambucinum*. Data show means of three replicates ± standard deviation. *^a^*, *^b^*: Significant differences between cultivars were detected *^A^*, *^B^*: Significant differences between storage stages (early and mid) were detected (*t*-test, *p*-value < 0.05).

		Early-Stage Potato Tubers		Mid-Stage Potato Tubers	
Mycotoxin	Cultivar	Days After Inoculation with *F. sambucinum*			
		10	40	10	40
T-2	Record	563.39 *^aA^* ± 232.43	44.07 *^aA^* ± 0	234.81 *^aA^* ± 37.55	ND
	Casablanca	357.91 *^aA^* ± 256.7	11.92 *^bA^* ± 0	110975.01 *^bB^* ± 41710.08	18.49 *^bA^* ± 0.02
HT-2	Record	293.59 *^aA^* ± 0	ND	60.18 *^aB^* ± 6.51	ND
	Casablanca	157.22 *^aA^* ± 0	8.01 *^bA^* ± 0	32584.1 *^bB^* ± 7938.67	18.01 *^Ab^* ± 0
DAS	Record	198.91 *^Aa^* ± 87.1	6.24 *^Aa^* ± 0.76	153.08 *^aA^* ± 29.22	1.93 *^aA^* ± 0
	Casablanca	175.33 *^Aa^* ± 0	9.57 *^Aa^* ± 0.68	37350.56 *^bB^* ± 10365.22	26.44 *^bA^* ± 0
15-AS	Record	213.67 *^aA^* ± 0	14.04 *^aA^* ± 2.7	42.00 *^aB^* ± 8.33	ND
	Casablanca	ND	30.67 *^bA^* ± 0	30537.21 *^bB^* ± 352.97	65.75 *^bA^* ± 0
Neosolaniol	Record	48.42 *^aA^* ± 0	ND	8.62 *^Ba^* ± 0.25	ND
	Casablanca	16.89 *^bA^* ± 0	ND	6219.92 *^Bb^* ± 1809.04	ND
Beauvericin	Record	ND	ND	169.53 *^aA^* ± 0	ND
	Casablanca	78.78 *^bA^* ± 0	ND	550.34 *^bB^* ± 64.93	ND

ND: Not detected.

## Data Availability

Data supporting this study are openly available from CORD, at this link: https://doi.org/10.57996/cran.ceres-2594.

## Supplementary Materials

The following supporting information can be downloaded at https://www.mdpi.com/article/10.3390/toxins16100414/s1, Figure S1: Effect of temperature (5, 10, 15°C) and a_w_ (0.97, 0.99) on F. sambucinum and F. oxysporum growth on colony morphology of *F. sambucinum* and *F. oxysporum* after 10 days of storage on potato-based media (NPDA). Table S1: Mycotoxin, retention time, qualifier (Q1) and quantifier (Q3) and recovery information about the matrix effect (LOD and LOQ).
