# Supplementary material for: Effect of Temperature, Relative Humidity, and Incubation Time on the Mycotoxin Production by Fusarium spp. Responsible for Dry Rot in Potato Tubers
Source: Toxins (Basel). 2024 Sep 24;16(10):414. doi: 10.3390/toxins16100414 (PMC11511537; doi:10.3390/toxins16100414)
Supplement: Supplementary file 1 [file toxins-16-00414-s001.zip › toxins-3157982-supplementary.pdf]

toxins

Supplementary Materials

Effect of Temperature, Relative Humidity, and Incubation Time on the Mycotoxin Production by *Fusarium* spp. Responsible for Dry Rot in Potato Tubers

Maria Gutiérrez-Pozo, Carol Verheecke-Vaessen, Sofia Kourmpetli [2], Leon A. Terry and Angel Medina

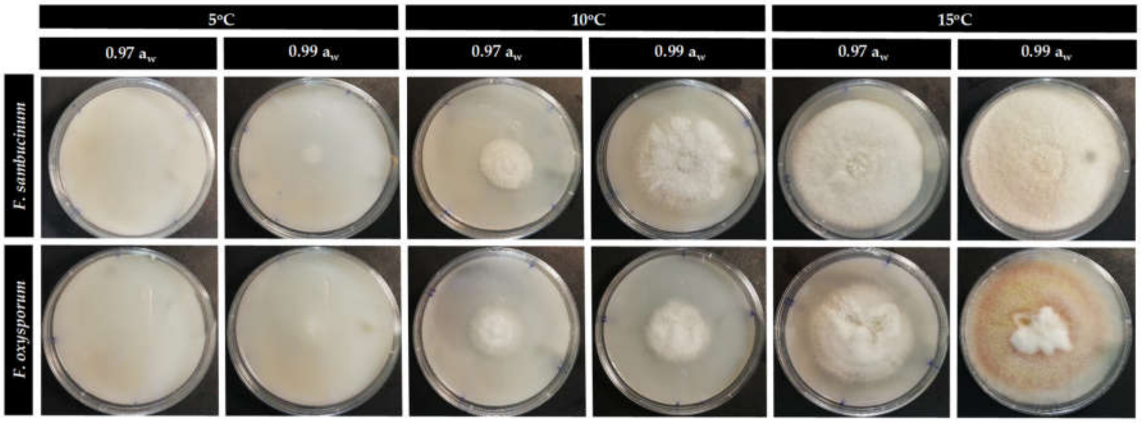

Figure S1. Effect of temperature (5, 10, 15°C) and a_w (0.97, 0.99) on *F. sambucinum* and *F. oxysporum* growth on colony morphology of *F. sambucinum* and *F. oxysporum* after 10 days of storage on potato-based media (NPDA).

Table S1. Mycotoxin, retention time, qualifier (Q1) and quantifier (Q3) and recovery information about the matrix effect (LOD and LOQ).

Mycotoxin	Retention time (min)	m/z Q1	DP (V)	m/z Q3	CE (V)	CXP (V)	Recovery (%)	LOD	LOQ
**T-2**	6.73	484.3	57/27	215.2/185.1	29/33	17/11	87.80	0.44	1.47
**HT-2**	6.17	447.4	131/50	345.1/323.2	15/29	20/16	102.12	0.40	1.35
**3-Acetyldeoxynivalenol**	4.36	397.3	-70	59.2/307.1	-38/-20	-8/-7	97.35	3.48	11.61
**15-Acetyldeoxynivalenol**	4.38	397.1	91	137.2/321.2	17/13	8/18	89.82	0.41	1.36
**Zearalenone**	6.92	317.1	-110	175.0/121.1	-34/-42	-13/-8	111.30	0.1	0.34
**Nivalenol**	1.01	371.1	-75	59.1/281.1	-42/-22	-14/-19	104.16	10.71	46.68
**Deoxynivalenol**	1.78	355.1	-70	59.2/265.2	-40/-22	-13/-10	78.83	14.9	49.65
**Alternariol**	5.67	257.0	-100	213.0/215.0	-34/-36	-11	130.74	0.27	0.91
**Fumonisin B1**	6.40	722.5	121	352.3/334.4	55/57	12/4	82.80	133.54	445.14
**Fumonisin B2**	7.88	706.5	126	336.4/318.4	59/51	8/2	84.12	51.18	170.6
**Diacetoxyscirpenol**	5.51	384.2	71	307.0/246.9	15/21	28/14	99.12	0.91	3.02
**Neosolaniol**	3.99	400.2	76	215.0/185.0	25/29	12/14	92.04	1.45	4.85
**Beauvericin**	11.03	801.5	116/191	244.2/384.4	47/73	12/10	117.97	0.59	1.95
**15-Acetoxyscirpenol**	5.00	342.2	71	265.1/307.2	13	26/8	100.93	2.11	7.02
